# Mesoscopic Calculation of Single Mismatches in RNA/DNA Hybrids: Strong Hydrogen Bonds of dTrG Affect CRISPR Off-Target Binding

**DOI:** 10.1021/acs.jpcb.6c01938

**Published:** 2026-06-19

**Authors:** Maria Izabel Muniz, Erik de Oliveira Martins, Thomas Carzaniga, Stefano Marni, Marco Buscaglia, Gerald Weber

**Affiliations:** † Dipartimento di Biotecnologie Mediche e Medicina Traslazionale, Università degli Studi di Milano, 20054 Segrate, MI, Italy; ‡ Instituto Federal de Educação, Ciência e Tecnologia de Minas Gerais, Campus Ribeirão das Neves, Ribeirão das Neves, MG 33805-488, Brazil; § Departamento de Física, 28114 Universidade Federal de Minas Gerais, Belo Horizonte, MG 31270-901, Brazil

## Abstract

In nature, complementary DNA–RNA hybrids occur in several biological contexts, mainly related to transcription, replication, and gene regulation. Despite their importance, mismatches in DNA-RNA hybrids are not as well characterized as in the case of DNA–DNA or RNA–RNA pairing. Of particular concern are strong mismatches between DNA and RNA strands that may favor off-target binding in CRISPR-Cas gene editing. Here, we investigate the effect of single internal mismatches on the thermal stability of RNA/DNA sequences using a mesoscopic approach based on published melting temperatures, as well as new measurements, to estimate hydrogen bonds and stacking interaction potentials. Our results show that only dTrG base pairs have substantial hydrogen bonding, even larger than the Watson–Crick dTrA pair, while all others are negligible. However, we show that in many cases stacking provides some stability to the otherwise weaker mismatches. Our results provide an explanation for the stability of dTrG as well as why it has CRISPR editing efficiencies similar to those of perfect matches.

## Graphical abstract

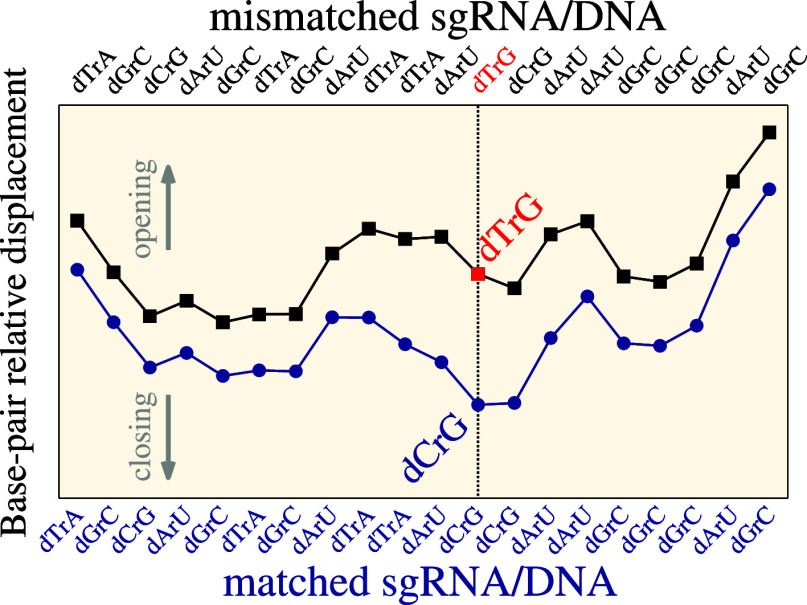

## Introduction

Mismatched base pairs in DNA and RNA are noncomplementary base pairs, that is, their pairing is neither CG nor AT/AU. A historically famous example is the wobble base pair GU in RNA, first proposed by Crick in 1966,^1^ which is the most common noncomplementary base pair in RNA duplexes. Differently from the CG and AU, the GU base pairs have a varying number of hydrogen bonds depending on their flanking neighbors,^2^ and their stacking interactions are equally dependent on sequence context. This variability is also common for all types of mismatched base pairs in DNA.^3^

DNA/DNA and RNA/RNA mismatches are responsible for genetic mutations, especially single nucleotide polymorphisms (SNPs), leading to replication errors^4–6^ or misincorporation in DNA.^7,8^ The structural stability provided by complementary base pairs is essential for the effective biological functions of all organisms, to the point that they have an extensive mismatch repair (MMR) mechanism.^9^ The study of MMR proteins is a guide to investigating diseases, since the loss of these proteins is directly associated with polyposis and tumors,^10^ and an excess of mismatches can be a source of diseases. In addition to their biological relevance, mismatches play a role in technological applications. For instance, in PCR, mismatches at probe positions are occasionally unavoidable, particularly for RNA viruses, requiring frequent updates to probe sets.^11,12^ On the other hand, they can also be an asset as they mark positions of probes that detect SNPs.^13,14^ In diagnostic and analytical technologies targeting miRNA or other RNA biomarkers, mismatches with DNA probes can directly affect the specificity of detection.^15^ DNA/RNA mismatches represent an important problem for CRISPR gene editing as they can cause off-target hybridization. In CRISPR, a short guide RNA (sgRNA) targets a specific complementary DNA section in the genome by forming an R-loop. If this section instead contains a stable mismatch, binding may occur to an unintended target. DNA/RNA mismatches also play an important role in RNA polymerase when misincorporation occurs. In this context, it was found that the dTrG mispair has the most common error, over 2 orders of magnitude larger than any other type of mismatch.^16^

Complementary base pairs tend to have a similar stability. For instance, as a result of strong hydrogen bonding, CG always has the highest stability for DNA,^17^ RNA,^18^ as well as for the hybrid DNA/RNA.^19^ In contrast, for mismatches, there is no such similarity. For example, the known stability of a GA mismatch in DNA is completely different when in RNA.^20^ We can only establish a rule of thumb, which is that mismatches destabilize the duplex in most cases, therefore lowering the melting temperature. The problem with this rule is the exceptions. For instance, we know that some types of mismatches in DNA can be surprisingly stable, either due to increased hydrogen bonds or due to strong stacking.^3^ In other words, it is not really possible to infer the mismatch properties in one type of oligonucleotide by comparing them to another type. Therefore, even though the stabilities of mismatches in DNA are now well established,^3,21^ they can not be extrapolated to the hybrid DNA/RNA.

For DNA/RNA mismatches, little is known about their structural characteristics, except for dTrG, where NMR measurements have shown that it is likely hydrogen-bonded.^22^ The dTrG is well-known for being the most stable DNA/RNA mismatch,^23–25^ and Raman measurements found that duplexes containing dTrG had almost no influence over the hybrid duplex geometry.^26^ The stability of dTrG was related to off-target binding in CRISPR-Cas9.^27–33^ However, for the remaining 11 types of DNA/RNA mismatched base pairs, very little is known regarding their hydrogen bonds and stacking interactions. Here, we apply our mesoscopic model to DNA/RNA melting temperatures in an attempt to fill this knowledge gap.

To obtain information about mismatch base pairs from the melting temperatures of duplexes, a theoretical framework is required. Currently, there are two main models that can be used for this purpose: the nearest-neighbors (NN) model^34^ and a mesoscopic approach based on the Peyrard–Bishop (PB) model.^17,35^ These models provide different information about the base pairs. The NN model results in enthalpies and entropies that are straightforward to use but do not reveal much about the intramolecular interactions.^36^ In contrast, the PB model provides detailed hydrogen bond and stacking potentials but is mathematically complex and requires specialized software.^37^ The PB model is now well-established as a predictor of hydrogen bonding after being applied to a large variety of duplex systems. After our first studies with complementary DNA^17^ and RNA,^2,18^ we were able to apply and validate the method for many other types of oligonucleotides, including artificial nucleic acids^38,39^ and metal-mediated DNA.^40^ DNA mismatches were studied with both models by our group.^3,41^ For DNA/RNA mismatches, several studies applied the NN model.^23,24,42,43^ However, there is presently no mesoscopic analysis for DNA/RNA; only complementary base pairs were calculated.^19^ Here, we present the calculations for a complete set of single mismatches in DNA/RNA with the mesoscopic PB model.

## Materials and Methods

### The Peyrard–Bishop Model

The statistical physics Peyrard–Bishop (PB) model describes how the double-stranded helix denatures by using interaction potentials, and the Hamiltonian is defined as

$$H=\underset{i}{\sum }\frac{p_{i}^{2}}{2m}+W\left(y_{i},y_{i+1}\right)+V\left(y_{i}\right)$$ 1

where the first term is the kinetic energy associated with the transverse displacement of the *i* ^th^ base pair, *W*(*y*_*i*_, *y*_*i*+1_) is the harmonic potential to describe the stacking interactions along the monomers, *V* (*y*_*i*_) is the Morse potential to simulate the hydrogen bonding between the monomers, and *y*_*i*_ represents the base pair displacements.

The representation of the on-site interaction by Morse potential is given by,

$$V\left(y_{i}\right)=D{\left(e^{-y_{i}/\lambda }-1\right)}^{2}$$ 2

where *D* is the dissociation constant and λ controls the potential width. The stacking interaction is

$$W\left(y_{i},y_{i+1}\right)=\frac{k_{i,i+1}}{2}\left(y_{i}^{2}-2y_{i}y_{i+1}\cos\;\theta +y_{i+1}^{2}\right)$$ 3

where *k* is the elastic constant and θ is the small twist angle between neighboring base pairs. The small angle was introduced to avoid numerical divergences in the partition function integral;^44^ we used θ = 0.01 rad.

The Hamiltonian is used to calculate the classic partition function over *N* base pairs,

$$Z_{y}=\int dy_{1}\int dy_{2}\cdot \cdot \cdot \int dy_{N}\overset{N}{\underset{i=1}{\prod }}e^{-\beta H}$$ 4

where β = 1/*k*_B_ *T* is the Boltzmann factor, *k*_B_ is the Boltzmann constant, and *T* is the absolute temperature. For the integration of eq 4, we used a Gauss-Legendre numerical method with 400 points over an interval of −0.1 to 20.0 nm.

From the partition function, eq 4, we calculate a melting index τ_*i*_ ^44^ to predict melting temperatures that are dependent on the structure of the sequence and proportional to the measured melting temperatures:

$$T_{i}^{\prime }\left(P\right)=a_{0}+a_{1}\tau_{i}\left(P\right)$$ 5

where *T*_*i*_′ is the predicted melting temperature, *P* is the set of tentative parameters, and *a*_0,1_ are linear regression coefficients. The set of tentative parameters *P* is comprised of a Morse potential *D* for each type of hydrogen bond and an elastic constant *k* for each type of nearest-neighbor stacking configuration.

To investigate a particular position in the sequences, we used the model to calculate the average displacement,

$$\left\langle y_{n}\right\rangle =\int dy_{1}\int dy_{2}\cdot \cdot \cdot \int dy_{N}y_{n}\overset{N}{\underset{i=1}{\prod }}e^{-\beta H}$$ 6

where *y*_*n*_ corresponds to an *n*-th position in the sequence for an absolute temperature *T*. That temperature is unrelated to the melting temperature of the data set, and the integration is realized over the same interval of the partition function.

### Melting Temperature Data Set

We used the following published melting temperatures: 57 sequences from Sugimoto et al.,^23^ 50 sequences from Watkins et al.,^42^ and 99 sequences from a preprint by Xiang et al.^43^ Of these, 193 sequences contain single DNA/RNA mismatches, and the remaining sequences contain only canonical base pairs. We also included measurements for sequences containing deoxyuridine (dU) in the DNA strand from ref 23. The temperatures were reportedly measured in a buffer containing 1 M NaCl, 10 mM Na_2_HPO_4_, and 1 mM Na_2_EDTA, adjusted to pH 7.0 and a strand concentration *C*_t_ =100 μM. Here, we recalculated these melting temperatures to 10 μM from the enthalpies and entropies obtained from the *T*_m_^–1^ × log *C*_t_ fit. Table S1 shows the sequences containing mismatches, and Table S2 shows the sequences containing only canonical base pairs.

In addition, 15 new sequences containing single mismatches were measured for nearest-neighbor configurations that were missing in earlier works.^23,42^ These new sequences were designed as variations of the canonical sequences from refs 23,42 , namely sequences C1, C6, and C12, as shown in Table S2. Several of these additional nearest-neighbor configurations were found to be very unstable and are shown in Table S3. They were measured at a total strand concentration of 10 μM, see Section S1. The experimental procedure is shown in Section S1, and the absorbance curves and analysis are shown in Figure S1.

### Notation

To unambiguously represent the DNA/RNA base pairs and nearest-neighbor configuration, we introduce the notation dA, dT, dC, and dG for DNA bases and rA, rU, rC, and rG for RNA bases. The specific case of deoxyuridine is written as dU. This allows us to write the canonical base pairs as dTrA, dArU, dGrC, and dCrG and the mismatches as dArA, dTrC, dGrU, and so forth. For the Morse potential, eq 2, the order of the base pair is not relevant; for instance, dTrA and rAdT represent the same hydrogen bond. For the nearest-neighbor configuration, we simply write the two base pairs in sequence, for example, dArA-dGrC, meaning a dArA mismatch followed by a dGrC canonical base pair. However, for the stacking potential of eq 3, the base pair order matters. For instance, dArA-dGrC is not the same as dArA-rCdG. The stacking equivalence is worked out by considering the duplex symmetry by taking into account the 5*′*→ 3*′* direction of the antiparallel strands. The following example shows how this is done for dTrG-dGrC
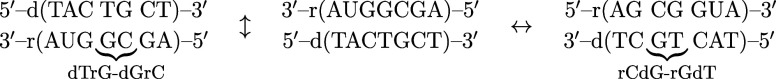


First, we swap the strands vertically, and next, we reverse them horizontally such that the upper strand is again in the 5*′*→ 3*′* direction. In this example, the symmetry equivalence indicates that the stacking configuration dTrG-dGrC is the same as rCdG-rGdT.

### Melting Temperature Optimization

To compare the predicted (*T*_*i*_′) and measured melting temperatures (*T*_*i*_), we use the parameter χ^2^ defined as the sum of the squares of their differences,^45^

$$\chi^{2}=\overset{N}{\underset{k=1}{\sum }}{\left[T_{i}-T_{i}^{\prime }\left(P_{k}\right)\right]}^{2}$$ 7

where *P*_*k*_ is the *k*th set of tentative parameters. The main goal is to minimize χ^2^ to ensure that the predicted temperatures are accurate and close to the experimental temperatures. In addition to eq 7, for a simpler analysis, we also calculate the average prediction difference,

$$\left\langle \Delta T\right\rangle =\frac{1}{N}\overset{N}{\underset{i=1}{\sum }}\left|T_{i}-T_{i}^{\prime }\left(P_{k}\right)\right|$$ 8

### Optimization Procedure

To minimize eq 7, we start by defining an optimal set of parameters *P*_m_ composed of 113 parameters, 13 Morse potentials (*D*), and 100 stacking interaction potentials (*k*). As the RNA/DNA canonical parameters were already calculated by Martins et al.,^19^ *P*_m_ contains only mismatch parameters. The minimization procedure is divided into three main steps, where we perform variations of the Morse and stacking potentials under different conditions. However, in this procedure, the optimization algorithm can occasionally reach a local minimum, which implies an incompatible set of parameters. To mitigate this limitation, each minimization step is carried out 100 times, where each one is a different initial set of parameters until it finds the best fit between the experimental and predicted data.

For the first step, we use the set of seed parameters *P*_*m*,seed_ shown in supporting material in Tables S4 and S5 for Morse and stacking interaction potentials, respectively. At each iteration (1–100), we select different initial parameters *P*_m,k_ randomly chosen within ±20% of their corresponding *P*_m,seed_. In the end, 100 sets of parameters *P*_m,k_ are calculated, and then by averaging them, we get a set P. Once the interactions are complete, we calculate the average of the 100 sets of parameters *P*_m,k_ to obtain a new final adjusted set *P*_m,F1_. In the second step, *P*_m,F1_ is used as the set of seed parameters, and the parameters are randomly chosen within ±10%. Considering the same approach as in the previous step, at the end of this step, a new adjusted final set *P*_m,F2_ is obtained. After two steps, the last one consists of validating *P*_m,F2_ including the uncertainty of the experimental measurements from Sugimoto et al.,^23^ Watkins et al.,^42^ and the new data set as ± 0.5 °C. The final set of Morse and stacking interaction parameters *P*_m,F3_, and their respective errors are shown in Tables S6 and S7 in supporting material. For *P*_m,F3_, the final merit factor and average prediction difference are χ^2^ = 2151 °C^2^ and ⟨Δ*T*⟩ = 2.25 °C, respectively.

## Results and Discussion

The first two sets of published melting temperatures for RNA/DNA mismatches, from Sugimoto et al.^23^ and Watkins et al.,^42^ contain 100 sequences, which do not cover all nearest-neighbor configurations. To cover the missing configurations, we measured 15 new sequences, which were designed as variations of the canonical sequences from refs 23,42 . The canonical sequences are shown in Table S2, and the new sequences are shown in Table S3. Some of the new sequences were found to be very unstable, having a melting temperature between 14 and 27 °C lower than their canonical counterparts. Later during our project, we became aware of the experimental data from Xiang et al.,^43^ also covering the missing nearest-neighbor configurations albeit with different sequences, which we added to our data set. Together with the new melting temperature measurements, the optimization was performed over a set of 221 RNA/DNA sequences, of which 208 contained a single mismatch, while the remaining contained only nonmismatched pairs. The number of unique combinations of base pairs and nearest-neighbors is shown in Tables S4 and S5, respectively. For nearest-neighbors, the configurations range between 2 and 7 unique stacking combinations, which are sufficient for establishing average parameters independent of sequence context.^3^ Since the mismatches increase the number of parameters to optimize, we had to consider 13 hydrogen bonding (parameter *D*) and 100 different stacking interactions (parameter *k*). It should be noted that this includes the non-natural dUrG base pair. Such a large number of parameters requires a considerable computational effort, which is the reason why we had to limit each round to 100 optimization steps. We performed three rounds of parameter optimization, the first two to refine the data and the last to estimate the influence of the experimental uncertainty. After the last round, the final average prediction difference ⟨Δ*T*⟩ was 2.25 °C. This is similar to the average prediction difference of 2.35 °C, which we obtained in a previous work for DNA mismatches.^3^

Figure 1, and also Table S6, shows the calculated Morse potentials *D*, which are related to hydrogen bonding.^35,44^ The only natural base pair with a Morse potential larger than 10 meV was dTrG. While the stability of dTrG is well-known,^23–25^ it is nevertheless remarkable that we found the hydrogen bonding to be stronger than for the canonical dTrA. Our results show that this stability is largely due to hydrogen bonding and is also consistent with structural observations from Szekely et al.^22^, which consider between two to three possible hydrogen bonds for this base pair. The non-natural base pair dUrG has the second largest Morse potential, yet well below the weakest canonical dArU base pair. The third highest, albeit at 6.6 meV, already very small, is dGrU, for which NMR measurements also attributed hydrogen bonding.^22^ It should be noted that dTrG, dUrG, and dGrU are of type pyrimidine-purine (dYrR). All remaining base pairs were found to have Morse potentials smaller than 5 meV, which is similar to what was found for the majority of DNA mismatches^3^ as well as for weakly bonded rGrU in RNA.^2^ The base pair dCrC stands out as having the lowest Morse potential of only 1.2 meV.

**Figure 1:**
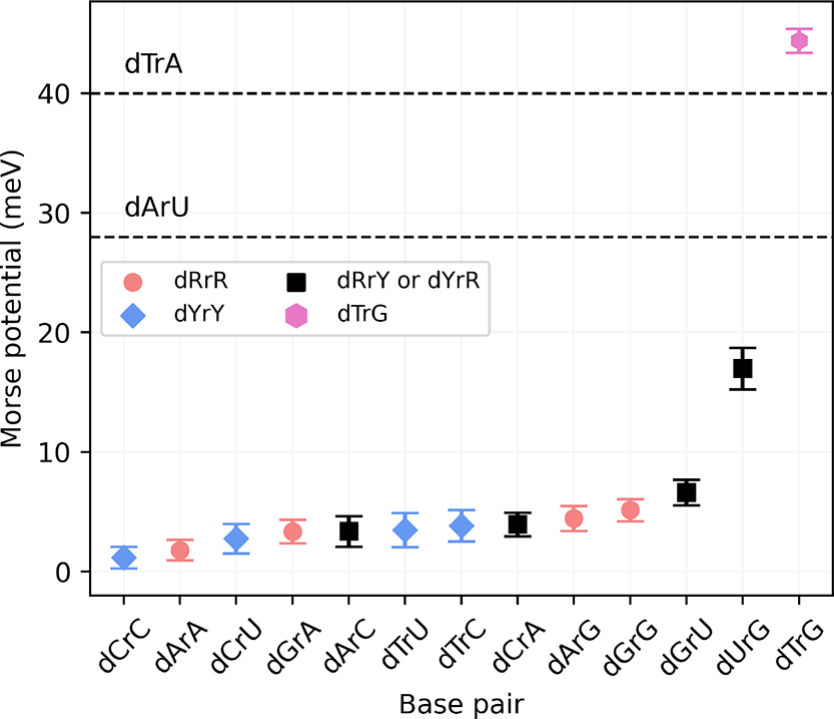
Average Morse potentials *D* for DNA/RNA mismatch base pairs in ascending order, where the colors correspond to the base configurations: purine–purine (dRrR, red bullets), pyrimidine-pyrimidine (dYrY, blue diamonds), and purine-pyrimidine (dRrY or dYrR, black boxes), and dTrG is highlighted as the pink hexagon. The error bars were obtained from the standard deviation of the last optimization round. The reference lines indicate the *D* values of the canonical base pairs dTrA and dArU from ref 19.

Figure 2 shows the optimized stacking interaction potentials (also in Table S7) for all nearest-neighbor combinations containing a single mismatched base pair. Compared to canonical DNA/RNA base pairs, where the highest stacking potential is 4.3 eV/nm^2^,^19^ a substantial number of mismatched stacking potentials are well above that value. This indicates that for many of these mismatches, the stabilization occurs mainly through stacking interactions since their Morse potentials are generally very weak. Some of these high stacking potentials are similar to those found for DNA mismatches involving purine–purine mismatches,^3^ some of which are well-known to be in a sheared stacking configuration. Given the similarity of those very large stacking configurations, we may speculate that those may contain some similar special arrangement. However, it should be noted that stacking usually plays a secondary role in oligonucleotide stabilization^46^ and that even strong stacking does not make up for a missing hydrogen bond. Unfortunately, the only structural study on DNA/RNA mismatches that we are aware^22^ does not provide enough information to allow a comparison to these results. Density functional theory (DFT) for hybrid DNA/RNA canonical dimers suggested that the hydrogen bonding capacity is increased by poor stacking,^47^ but if and how this would apply to mismatches is unclear. What is clear, however, is that steric effects seem to play a role. Canonical base pairs are formed by purine-pyrimidine bases and are generally isosteric to each other; that is, the distance between the C1′ atoms is the same,^48^ around 10.5 Å. Mismatches are not usually isosteric,^48^ especially if they are purine–purine or pyrimidine-pyrimidine. However, purine-pyrimidine mismatches will be close enough in size as not to cause large distortions to the helix. Therefore, one would expect better stacking for purine-pyrimidine mismatches than for purine–purine or pyrimidine-pyrimidine. This is what we observe from the stacking of purine-pyrimidine mismatches shown in Figure 2, where the majority of nearest-neighbor configurations are larger than 2 eV/nm^2^.

**Figure 2:**
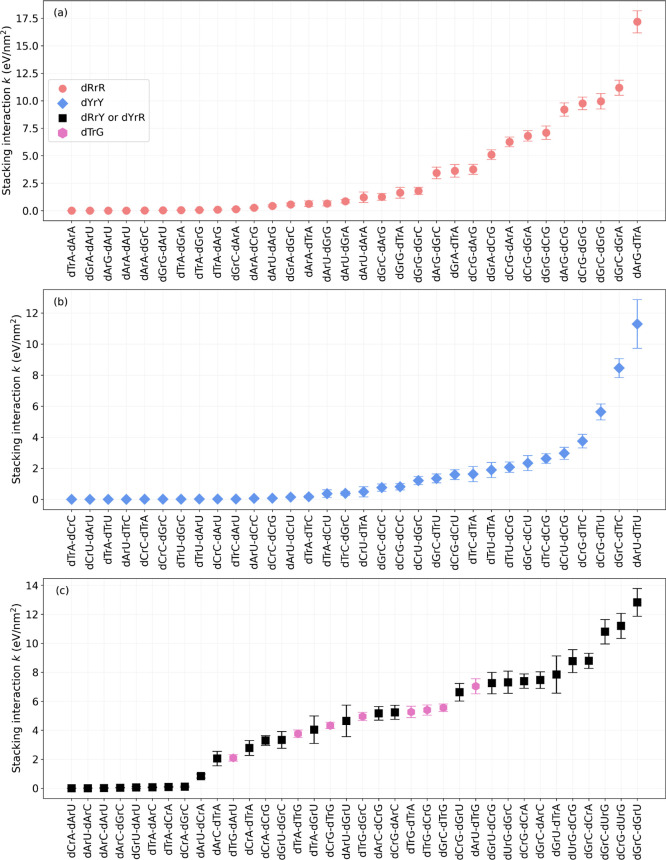
Average stacking potentials *k*, shown in ascending order for DNA/RNA mismatch nearest-neighbors for (a) purine–purine (dRrR, red bullets), (b) pyrimidine-pyrimidine (dYrY, blue diamonds), and (c) purine-pyrimidine (dRrY or dYrR, black boxes), where those containing dTrG are highlighted as pink hexagons. The colors and symbols are the same as for Figure 1. The error bars were obtained from the standard deviation of the last optimization round.

With the sole exception of one NMR measurement for dTrG and dGrU,^22^ we are not aware of enough structural studies of DNA/RNA mismatches for a comparative discussion of our results. On the other hand, there is an abundance of studies regarding the influence of DNA/RNA mismatches on CRISPR off-target editing efficiencies.^27–33,49,50^ Even though most of those focus on the distance of the mismatch location relative to the PAM site, many of them also analyze the type of mismatch, which could allow a correlation of our results with the editing efficiencies. For the comparison of the CRISPR-Cas9 editing efficiencies, we selected three different results from refs 27,30,51 , meeting the requirement of being available in terms of mismatch type. We start with the well-known work by Hsu et al.^27^ reporting cleaving efficiencies of Cas9 nucleases for over 700 single-guide RNA (sgRNA) targeting the human genome. Figure 3a shows the scatter plot of the average cleaving efficiencies of Cas9 nucleases for each type of mismatch^27^ compared to the calculated Morse potentials *D*. The linear regression of the scatter plot is shown as dashed lines. During the course of our analysis, we noticed that taking the logarithm of the Morse potential provides better correlation, likely due to the nonlinear nature of the PB model. Clearly, dTrG stands out in terms of both editing efficiency and highest *D*. The mismatch base pair dTrG is known for its stability,^23–25^ but this alone does not explain its exceptional cleaving efficiencies as there are other stable mismatches. In our case, dTrG is the only mismatch with a Morse potential similar to the canonical base pair dTrA; therefore, it seems plausible to attribute the exceptional cleaving efficiency to its hydrogen bonding. At the other extreme, the dCrC base pair also has both the weakest hydrogen bonding and the poorest editing efficiency. For the remaining mismatches, the correlation is less evident, but editing efficiencies are also dependent on distance relative to PAM sites, which may confound the correlation. Nevertheless, we found an *R* ^2^ = 0.66 by comparing the cleaving efficiencies with Morse potentials only, without any further adjustment. To place this *R* ^2^ value into perspective, several groups performed adjustments with the NN model Gibbs free energies, and the best correlation they obtained was *R* ^2^ = 0.64.^49,52–54^ Next, we compared our results to model parameters of CRISPR editing in bacteria from Hawkins et al.,^30^ shown in Figure 3b. Here again, dTrG is the highest and dCrC the lowest model parameters, as well as an *R* ^2^ = 0.66, identical to the results from^27^. A more recent analysis from Fu et al.,^51^ Figure 3c, also showed similar trends correlating to our results with *R* ^2^ = 0.72. Other works, not included in our analysis, like those of Jost et al.^31^ (see also van Gestel et al.^55^) and Doench et al.,^28^ also reported dTrG as having the highest efficiency^28,31^ , as well as dCrC as having the least sgRNA activity.^28^ The only different trend we found was from Zhang et al.,^33^ who reported on DNA/RNA mismatches in sgRNA comparing efficiency and off-target tolerance of the Cas9 variants HiFi and LZ3. They found that dTrG has only the second-highest efficiency, the highest being for dTrU instead. In their case, dCrC does not have the lowest efficiency ratio, with dGrA and dCrU having smaller ratios. The reason for this discrepancy is unclear, but one might speculate that this could be due to the fact that they are using variants of Cas9.

**Figure 3:**
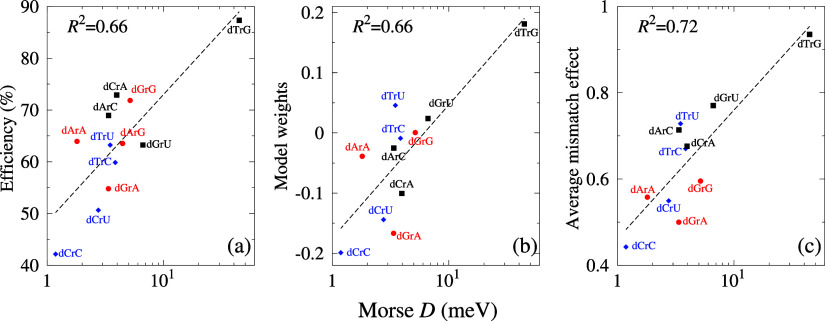
CRISPR editing efficiencies as a function of the logarithm of the Morse potential *D*. Panel (a) shows the editing efficiencies for single mismatches reported by Hsu et al.^27^ and panel (b) shows the model weights obtained by Hawkins et al.,^30^ and (c) shows the mismatch effects from Fu et al.^51^ Purine–purine base pairs are shown as red bullets, pyrimidine-pyrimidine as blue diamonds, and purine-pyrimidine as black boxes. *R* ^2^ is the coefficient of determination related to the linear regression, log_10_ *D* × *y*, shown as a dashed line in each panel. The colors and symbols are the same as for Figure 1.

Clearly, the strength of the mismatch Morse potential, and therefore the hydrogen bonding, plays a central role for the CRISPR off-target editing efficiencies. This would be a motivation to pursue a more detailed analysis of editing efficiencies by also taking into account the stacking interactions. With Morse potentials, the correlation of Figure 3 was straightforward to work out as the efficiency results from refs 27,30,51 were reported in terms of base pairs. Unfortunately, a similar comparison for stacking would require a completely new reanalysis from their raw data, which is presently beyond the scope of this work. Nevertheless, it is possible to discuss here certain aspects of how stacking, as well as the inherent nonlinearity of the model, may influence the overall stability of the duplex. For this discussion, we make use of the average displacement ⟨*y*_*n*_⟩, eq 6, which combines Morse potential and stacking interaction at base pair resolution. Larger ⟨*y*_*n*_⟩ means that the duplex is opening and therefore less stable at the *n*th position of the sequence. The combined effect of hydrogen bonding and stacking is illustrated in Figure 4, where we show three examples of sgRNA/DNA with and without a dTrG mismatch. Figure 4a shows a case where the dTrG causes very little difference in the average displacement compared to the fully matched duplex. A slightly larger, but very localized, opening is observed in Figure 4b. In contrast, a full disruption over the whole duplex is shown in Figure 4c. It may come as a surprise that this opening is not caused by the stacking on the dTrG mismatch but by the presence of a weakly hydrogen-bonded dArU neighbor. In fact, the stacking of the dTrG is the highest of these three examples. It should be noticed that, even for the fully matched sequence, there is already a pronounced opening around the highlighted dCrG site. When it is replaced by the much weaker dTrG, the entire duplex opens up. This example neatly illustrates that while hydrogen bonding provides a rough guide to the duplex stability, it is difficult to do the same with stacking. Only the full calculation of the average displacement conveys a complete picture of the mismatch destabilization.

**Figure 4:**
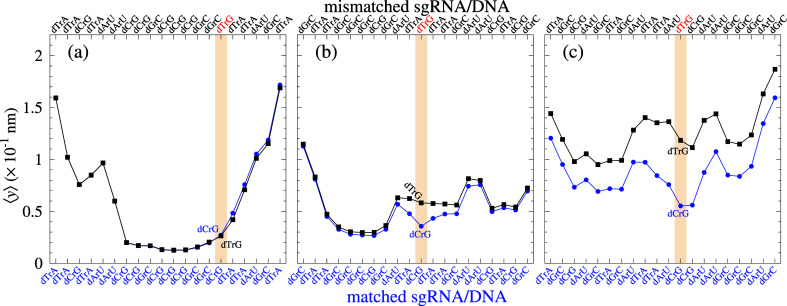
Average displacement profiles for sgRNA sequences matched to a DNA target. Blue bullets are for the fully matched sequence, and black boxes containing a single dTrG mismatch. The three sequences are from ref 33. Panels show the dTrG mismatch in three different situations: (a) with similar binding as a canonical dCrG, (b) with a moderate and localized opening, and (c) significantly disrupting the whole duplex. The shaded area indicates the position of the mismatch. Profiles were calculated at 240 K, eq 6, which is unrelated to the melting temperature.

## Conclusion

We presented a complete mesoscopic calculation of hydrogen bonds and stacking interactions for all 12 possible mismatch base pairs in DNA/RNA, as well as for the artificial dUrG base pair. Only the dTrG mismatch was found to have a hydrogen bond similar to the canonical dTrA base pair, while all others have very low or possibly no hydrogen bonding. Our new Morse potentials were correlated to three independent sets of CRISPR-Cas9 off-target editing efficiencies, and we found *R* ^2^ ranging between 0.66 and 0.72, which is better than correlations obtained from other models.^49,52–54^ In view of these results, the possible explanation for the exceptional off-target efficiency of dTrG appears to be its strong hydrogen bonding. At the other extreme, dCrC, which has the lowest hydrogen bond also correlates to the poorest off-target efficiencies. Our new parameters can be used for the calculation of duplex opening profiles in the presence of single DNA/RNA mismatches, allowing a detailed analysis of the hybridization patterns. We believe that our new parameters may be used to develop new methods of computational predictions of CRISPR editing efficiencies.

## Supplementary Material



## Data Availability

The parameters described in this work are included in the latest version of our free TfReg software,^37^ which can be used to verify our results. The software and the parameters are freely available at http://tinyurl.com/tfregufmg. Please see Supporting Information for additional download options.

## Notes

Section S1: experimental procedure of the additional melting temperature measurements; Figure S1: absorbance analysis; Tables S1 and S2: mismatch sequences and canonical sequences and their melting temperatures; Table S3: new sequences and their temperature measurements; Tables S4 and S5: seed parameters for Morse and stacking potentials; and Tables S6 and S7: optimized Morse and stacking parameters (PDF)
