# Supplementary material for: Mesoscopic Calculation of Single Mismatches in RNA/DNA Hybrids: Strong Hydrogen Bonds of dTrG Affect CRISPR Off-Target Binding
Source: J Phys Chem B. 2026 Jun 19;130(26):6569–76. doi: 10.1021/acs.jpcb.6c01938 (PMC13339645; doi:10.1021/acs.jpcb.6c01938)
Supplement: Supplementary file 1 [file jp6c01938_si_001.pdf]

Supporting information: Mesoscopic Calculation of Single Mismatches in RNA/DNA Hybrids: Strong Hydrogen Bonds of dTrG Affect CRISPR Off-target Binding

Maria Izabel Muniz,[1] Erik de Oliveira Martins,[2] Thomas Carzaniga,[1] Stefano Marni,[1] Marco Buscaglia,[1] and Gerald Weber[3]

[1] *Dipartimento di Biotecnologie Mediche e Medicina Traslazionale, Università degli Studi di Milano, 20090 Segrate, MI, Italy*[*]

[2] *Instituto Federal de Educação, Ciência e Tecnologia de Minas Gerais, Campus Ribeirão das Neves, 33805-488 Ribeirão das Neves, MG, Brazil*

[3] *Departamento de Física, Universidade Federal de Minas Gerais, 31270-901 Belo Horizonte, MG, Brazil*[†]

The software is available for multiple Linux distribution. All data files and software can be obtained from one of the following sources:

- https://bioinf.fisica.ufmg.br/software
- https://sites.google.com/site/geraldweberufmg/tfreg
- https://software.opensuse.org//download.html?project=home%3Adrgweber&package=TfReg
- http://download.opensuse.org/repositories/home:/drgweber/

LIST OF FIGURES

S1 Absorbance analysis. S3

LIST OF TABLES

S1 Sequences containing single mismatches used for optimization. S4
S2 Sequences containing only canonical base pairs. S7
S3 New DNA/RNA sequences containing single mismatches S7
S4 Seed Morse potential parameters and number of occurrences of mismatched base pair S8
S5 Seed harmonic potential parameters and number of occurrences of nearest-neighbor configurations S8
S6 Final Morse potential depths. S9
S7 Final harmonic potential coupling constants S10

* mariaizabelmc@gmail.com
† gweberbh@gmail.com

S1. USING UV HYPERCHROMICITY TO DETECT DNA-RNA HYBRID MELTING

Experiments were performed in melting buffer 1X (1 M NaCl, 10 mM Na_2HPO_4, 1 mM Na_2EDTA, pH 7.0) with a total nucleic acid concentration of 10 μM.

The overall degree of hybridization in DNA-RNA duplex was evaluated by measuring the absorbance A at the wavelength $\lambda = 260$ nm. A is obtained by averaging over an interval $\Delta\lambda = 3$ nm (259-261 nm). Experiments were performed with the Evolution 300 UV-Vis spectrophotometer from Thermo Scientific customized with a Quantum Northwest peltier hot/cold stage with hold temperature accuracy of ±0.05°C. Experiments have been performed with a standard quartz cuvette with optical path length $l = 1$ cm and a 1 °C/min heating and cooling temperature rate from -4 to 95 °C. In order to have significant statistics of the measurements, each sample was heated and cooled 4 times in this temperature range.

The absorbance values increase as the melting of the duplex occurs because of the hypochromic effect [1], so that it is possible to relate the $A(T)$ curves to the melting curves (T), i.e. the fraction of unpaired strands, as follows [2]:

$$A(T) = [1 - \rho_f(T)]A_{LT} + \rho_f(T)A_{HT}$$

where $A_{LT} = m_{LT}T + q_{LT}$ and $A_{HT} = m_{HT}T + q_{HT}$ represent the linear drift of the absorbance at low and high temperatures, respectively. With the hypothesis that the melting can be well represented by a two-state model, we used the expression of melting curve for a system composed by a complementary couple of strands with equimolar concentration [3]:

$$\rho_f(T) = \frac{2}{1 + \sqrt{1 + 2c * exp[-(\Delta H - T\Delta S)/RT]}}$$

where c is the total strand concentration (single stranded) and R the gas constant. ΔH and ΔS are the enthalpic and entropic contribution of the pairing that are free parameters of the fitting, together with m_{LT}, q_{LT}, m_{HT} and q_{HT}.

From the fit, we obtain $\rho_f(T)$ for the entire set of ramps, that are averaged and the melting temperature T_m of the sample is obtained as the temperature at which $\rho_f(T = T_m) = 0.5$. The choice to include the analytic expression of the melting curve in the fit was necessary because the low T_m of the dataset often prevented to directly observe the linear region $A_{LT}(T)$.

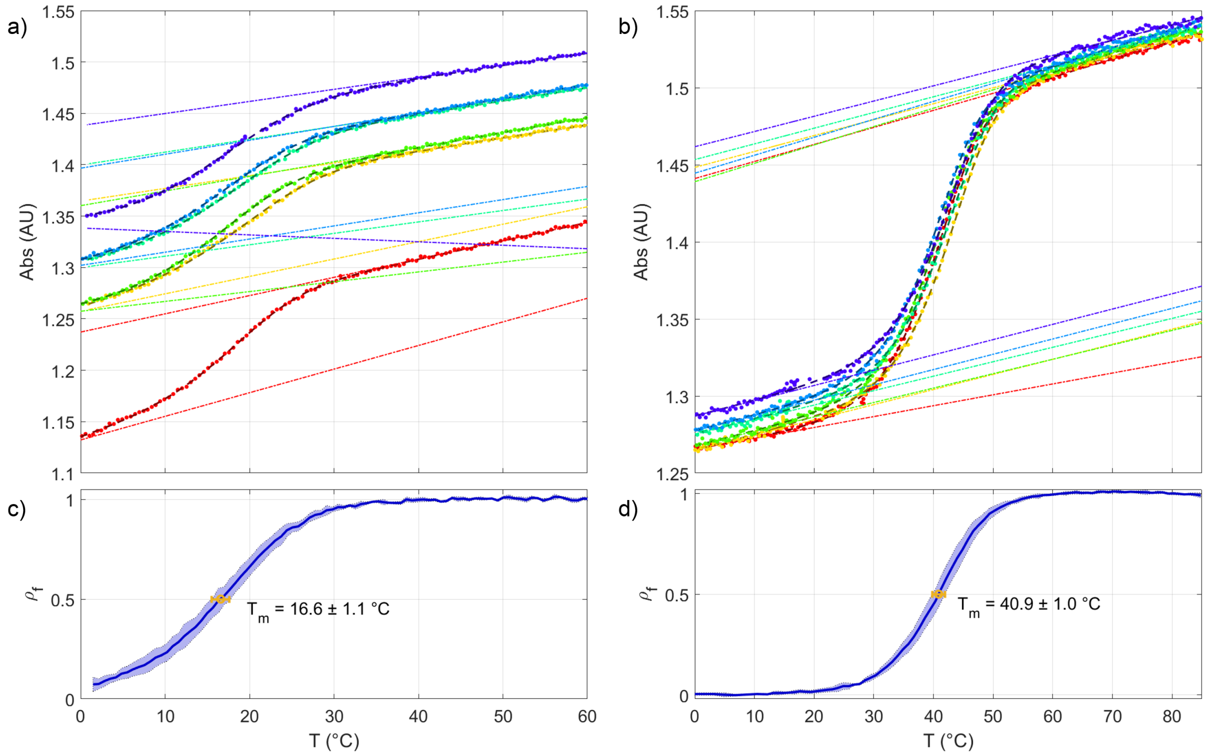

Figure S1. Absorbance analysis (a, b) and computed measured melting curves (c, d) for N15 mismatch (left) and a control canonical sequence C6 (right). Considering a duplex with low thermal stability, in panel (a) we notice that it is not possible to directly distinguish the plateau at low temperatures: the analysis described in the text allows to fit the entire curves, with still some fluctuations of the baselines. The best curves are then selected to compute, that is shown in panel (c). In panel (b), we notice that the melting of well paired duplex enables a higher quality analysis of, so that all the curves are considered for the computation, shown in panel (d).

Table S1. 193 DNA/RNA sequences containing single mismatches used for optimization . Shown are the primary structures with the upper strand in 5′ → 3′ direction, the measured melting temperatures (T_i) from Refs. [4–6], and their melting temperatures (T_i') predicted with our new parameters. All temperatures are in °C. The mismatch positions are highlighted in red.

Ref.	Sequence	T_i	T_i'	Ref.	Sequence	T_i	T_i'	Ref.	Sequence	T_i	T_i'
[4]	r(AAGCAGUAG)<br>d(TTCGACATC)	22.03	24.17	[4]	r(UCACACUAG)<br>d(AGTGAGATC)	10.6	10.79	[4]	r(UGAGAGUAC)<br>d(ACTCACATG)	28.11	23.94
[4]	r(UUGGACACC)<br>d(AACCAGTGG)	22.48	23.85	[4]	r(AAGCAGUAG)<br>d(TTCGCCATC)	24.81	26.25	[4]	r(UCACACUAG)<br>d(AGTGCGATC)	16.52	19.46
[4]	r(UGAGAGUAC)<br>d(ACTCCCATG)	32.32	29.07	[4]	r(UUGGACACC)<br>d(AACCCGTGG)	30.8	34.79	[4]	r(AAGCAGUAG)<br>d(TTCGGCATC)	25.63	27.25
[4]	r(UCACACUAG)<br>d(AGTGGGATC)	19.87	22.18	[4]	r(UGAGAGUAC)<br>d(ACTCGCATG)	32.21	29.98	[4]	r(UUGGACACC)<br>d(AACCGGTGG)	35.01	37.19
[4]	r(AAGCCGUAG)<br>d(TTCGACATC)	22.42	23.91	[4]	r(UGAGCGUAC)<br>d(ACTCACATG)	27.52	28.94	[4]	r(AAGCCGUAG)<br>d(TTCGTCATC)	24.19	24.56
[4]	r(AAGCGGUAG)<br>d(TTCGACATC)	24.97	28.02	[4]	r(UCACGCUAG)<br>d(AGTGAGATC)	15.61	18.41	[4]	r(UGAGGGUAC)<br>d(ACTCACATG)	32.81	30.74
[4]	r(UUGGGCACC)<br>d(AACCAGTGG)	31.43	34.03	[4]	r(AAGCGGUAG)<br>d(TTCGGCATC)	28.7	31.06	[4]	r(UCACGCUAG)<br>d(AGTGGGATC)	24.58	23.43
[4]	r(UGAGGGUAC)<br>d(ACTCGCATG)	34.81	33.63	[4]	r(UUGGGCACC)<br>d(AACCGGTGG)	38.33	38.32	[4]	r(AAGCGGUAG)<br>d(TTCGTCATC)	40.05	39.02
[4]	r(UCACGCUAG)<br>d(AGTGTGATC)	31.64	32.99	[4]	r(UGAGGGUAC)<br>d(ACTCTCATG)	40.5	39.01	[4]	r(UUGGGCACC)<br>d(AACCTGTGG)	45.19	44.47
[4]	r(UCUACGCAG)<br>d(AGATGTGTC)	29.84	32.98	[4]	r(UUAGACUGGC)<br>d(AATTTGACCG)	37.2	38.75	[4]	r(UUAAGCUGGC)<br>d(AATTTGACCG)	41.5	39.06
[4]	r(UUAACGUGGC)<br>d(AATTGTACCG)	39.51	41.07	[4]	r(UUAACUGGGC)<br>d(AATTGATCCG)	38.1	37.89	[4]	r(AAGCUGUAG)<br>d(TTCGCCATC)	23.6	23.62
[4]	r(UCACUCUAG)<br>d(AGTGCGATC)	14.04	17.21	[4]	r(UGAGUGUAC)<br>d(ACTCCCATG)	26.5	23.72	[4]	r(UUGGUCACC)<br>d(AACCCGTGG)	26.42	30.32
[4]	r(AAGCUGUAG)<br>d(TTCGGCATC)	30.7	30.58	[4]	r(UCACUCUAG)<br>d(AGTGGGATC)	26.3	26.61	[4]	r(UGAGUGUAC)<br>d(ACTCGCATG)	32.97	32.14
[4]	r(UUGGUCACC)<br>d(AACCGGTGG)	38.24	40.12	[4]	r(UGUAGUGAC)<br>d(ACATCGCTG)	33.97	32.14	[4]	r(UUAUACUGGC)<br>d(AATGTGACCG)	34.53	34.19
[4]	r(UUAAUCUGGC)<br>d(AATTGGACCG)	35.16	36.23	[4]	r(UUAACUUGGC)<br>d(AATTGGACCG)	32.26	31.92	[4]	r(UUAACUUGGC)<br>d(AATTGAGCCG)	31.68	31.23
[4]	r(UCACUCUAG)<br>d(AGTGTGATC)	14.42	12.75	[4]	r(UGAGUGUAC)<br>d(ACTCTCATG)	28.72	26.58	[4]	r(UUGGUCACC)<br>d(AACCTGTGG)	27.54	28.65
[4]	r(AAGCGGUAG)<br>d(TTCGUCATC)	37.75	38.73	[4]	r(UCACGCUAG)<br>d(AGTGUGATC)	31.84	30.93	[4]	r(UGAGGGUAC)<br>d(ACTCUCATG)	39.94	38.94
[4]	r(UUGGGCACC)<br>d(AACCUGTGG)	41.91	42.88	[5]	r(AGGUAAGGU)<br>d(TCCAATCCA)	24.52	33.78	[5]	r(CCACAACAGAG)<br>d(GGTGTAGTCTC)	38.16	38.29
[5]	r(GUCCUAGCUCA)<br>d(CAGGAACGAGT)	34.53	36.48	[5]	r(CAUGAAGCUAC)<br>d(GTACTACGATG)	35.89	36.22	[5]	r(GAGACAACACC)<br>d(CTCTGATGTGG)	39.07	39.87
[5]	r(CGAGGAAUGGC)<br>d(GCTCCATACCG)	44.15	49.36	[5]	r(GAACUACUGUG)<br>d(CTTGAAGACAC)	27.72	27.46	[5]	r(GCUCAAACCCG)<br>d(CGAGTATGGGC)	38.92	39.92
[5]	r(GCCGUAUCAAC)<br>d(CGGCAAAGTTG)	36.05	36.41	[5]	r(CGCAGAUCCAC)<br>d(GCGTCAAGGTG)	40.99	41.71	[5]	r(AGGUCAGGU)<br>d(TCCACTCCA)	23.71	33.82
[5]	r(GGACCGACG)<br>d(CCTGCCTGC)	30.91	33.27	[5]	r(CCACACCAGAG)<br>d(GGTGTCGTCTC)	33.53	37.22	[5]	r(GUCCUCGCUCA)<br>d(CAGGACCGAGT)	32.34	32.26
[5]	r(CAUGACGCUAC)<br>d(GTACTCCGATG)	32.81	29.25	[5]	r(GAGACCACACC)<br>d(CTCTGCTGTGG)	36.28	39.79	[5]	r(CGAGGCAUGGC)<br>d(GCTCCCTACCG)	44.04	48.85
[5]	r(GGCGACUGAUG)<br>d(CCGCTCACTAC)	36.36	41.37	[5]	r(GACUGCCUCCA)<br>d(CTGACCGAGGT)	34.92	32.21	[5]	r(CGAGCGAUGGC)<br>d(GCTCCCTACCG)	43.82	45.72
[5]	r(CGCAGCUCCAC)<br>d(GCGTCCAGGTG)	39.58	39.44	[5]	r(AGGUGAGGU)<br>d(TCCAGTCCA)	32.7	34.26	[5]	r(CCACAGCAGAG)<br>d(GGTGTGGTCTC)	46.04	46.72
[5]	r(GUCCUGGCUCA)<br>d(CAGGAGCGAGT)	39.04	39.38	[5]	r(CAUGAGGCUAC)<br>d(GTACTGCGATG)	41.53	39.64	[5]	r(GAGACGACACC)<br>d(CTCTGGTGTGG)	44.19	43.29
[5]	r(CGAGGGAUGGC)<br>d(GCTCCGTACCG)	53.7	55.62	[5]	r(GAACUGCUGUG)<br>d(CTTGAGGACAC)	34.7	35.37	[5]	r(GGCGAGUGAUG)<br>d(CCGCTGACTAC)	43.71	45.61

(continues on next page)

(S1 continued from previous page)

Ref.	Sequence	T_i	T'_i	Ref.	Sequence	T_i	T'_i	Ref.	Sequence	T_i	T'_i
[5]	r(GCCACGUCACG)<br>d(CGGTGGAGTGC)	44.45	44.13	[5]	r(CGCAGGUCCAC)<br>d(GCGTCGAGGTG)	47.98	47.53	[5]	r(AGGUUAGGU)<br>d(TCCATTCCA)	26.9	33.84
[5]	r(GGACUGACG)<br>d(CCTGTCTGC)	34.25	37.7	[5]	r(CCACAUCAGAG)<br>d(GGTGTTGTCTC)	40.94	41.65	[5]	r(GUCCUUGCUCA)<br>d(CAGGATCGAGT)	36.53	36.44
[5]	r(CAUGAUGCUAC)<br>d(GTACTTCGATG)	38.02	37.08	[5]	r(GAGACUACACC)<br>d(CTCTGTTGTGG)	40.33	39.88	[5]	r(CGAGGUAUGGC)<br>d(GCTCCTTACCG)	49.83	51.45
[5]	r(GAACUUCUGUG)<br>d(CTTGATGACAC)	29.14	29.51	[5]	r(GCUCAUACCCG)<br>d(CGAGTTTGGGC)	39.95	40.83	[5]	r(GCCGUUUCAAC)<br>d(CGGCATAGTTG)	39.62	39.98
[5]	r(GCCACUUCACG)<br>d(CGGTGTAGTGC)	43.73	43.39	[6]	r(GUUGAACCUAC)<br>d(CAACTCGGATG)	36.4	34.94	[6]	r(GUGUAAGUACC)<br>d(CACATCCATGG)	34.19	35.68
[6]	r(GAUCAAUCCAC)<br>d(CTAGTCAGGTG)	35.43	32.77	[6]	r(CGACCAACUUG)<br>d(GCTGGCTGAAC)	34.79	33.59	[6]	r(UCACACGAUAA)<br>d(AGTGCGCTATT)	32.54	29.69
[6]	r(GCGUCAGUCAC)<br>d(CGCAGCCAGTG)	43.7	42.14	[6]	r(CUAACAUGAGG)<br>d(GATTGCACTCC)	34.11	35.05	[6]	r(GUAGGAACAUG)<br>d(CATCCCTGTAC)	38.59	37.88
[6]	r(CAGUGACCAAG)<br>d(GTCACCGGTTC)	43.1	44.6	[6]	r(CCUAGAGCAUG)<br>d(GGATCCCGTAC)	40.97	42.76	[6]	r(CAGUGAUCAAG)<br>d(GTCACCAGTTC)	37.59	34.64
[6]	r(GUGUAAGUACC)<br>d(CACACTCATGG)	33.26	30.48	[6]	r(CAUGUACAGUG)<br>d(GTACACGTCAC)	37.82	33.27	[6]	r(GCACUAUACCG)<br>d(CGTGACATGGC)	35.42	36.41
[6]	r(GAGCAAACGUC)<br>d(CTCGTGTGCAG)	41.17	41.0	[6]	r(GUUGAACCUAC)<br>d(CAACTGGGATG)	36.23	36.4	[6]	r(GAUCAAUCCAC)<br>d(CTAGTGAGGTG)	35.68	33.16
[6]	r(CGACCAACUUG)<br>d(GCTGGGTGAAC)	35.01	34.26	[6]	r(CAUUCACUACG)<br>d(GTAAGGGATGC)	31.51	28.88	[6]	r(GCGUCAGUCAC)<br>d(CGCAGGCAGTG)	44.78	43.27
[6]	r(CUAACAUGAGG)<br>d(GATTGGACTCC)	35.23	36.46	[6]	r(GUAGGAACAUG)<br>d(CATCCGTGTAC)	38.91	38.68	[6]	r(CAGUGACCAAG)<br>d(GTCACGGGTTC)	45.07	46.82
[6]	r(CCUAGAGCAUG)<br>d(GGATCGCGTAC)	41.52	43.58	[6]	r(CAGUGAUCAAG)<br>d(GTCACGAGTTC)	38.97	35.95	[6]	r(GUGUAAGUACC)<br>d(CACAGTCATGG)	33.85	30.39
[6]	r(CAUGUACAGUG)<br>d(GTACAGGTCAC)	38.0	34.4	[6]	r(GCACUAUACCG)<br>d(CGTGAGATGGC)	36.64	36.43	[6]	r(GCACUAUACCG)<br>d(CGTGATCTGGC)	35.78	38.64
[6]	r(GAUCAAUCCAC)<br>d(CTAGTTCGGTG)	35.08	32.43	[6]	r(CUAACAUGAGG)<br>d(GATTGTCCTCC)	35.47	37.46	[6]	r(UAAGAUUGGUA)<br>d(ATTCTCACCAT)	31.17	28.13
[6]	r(UGGUCUGUAGA)<br>d(ACCAGCCATCT)	36.7	37.55	[6]	r(GAACCUCUCUA)<br>d(CTTGGCGAGAT)	32.97	30.2	[6]	r(GGAACUUCAUU)<br>d(CCTTGCAGTAA)	31.28	29.92
[6]	r(CAUGUACAGUG)<br>d(GTACCTGTCAC)	37.8	34.99	[6]	r(UACAGUCCAUG)<br>d(ATGTCCGGTAC)	36.49	34.85	[6]	r(AACGGUGCGAA)<br>d(TTGCCCCGCTT)	44.72	49.31
[6]	r(CUACGUUGCAA)<br>d(GATGCCACGTT)	33.82	33.51	[6]	r(UAAGAUUGGUA)<br>d(ATTCTACCCAT)	30.62	27.28	[6]	r(AGUCUUUCAGG)<br>d(TCAGACAGTCC)	30.85	34.87
[6]	r(GAUCAAUCCAC)<br>d(CTAGTTGGGTG)	45.11	42.73	[6]	r(CUAACAUGAGG)<br>d(GATTGTGCTCC)	42.15	45.65	[6]	r(UAAGAUUGGUA)<br>d(ATTCTGACCAT)	36.47	34.87
[6]	r(UGGUCUGUAGA)<br>d(ACCAGGCATCT)	41.28	43.54	[6]	r(GGAACUUCAUU)<br>d(CCTTGGAGTAA)	36.69	34.9	[6]	r(CAUGUACAGUG)<br>d(GTACGTGTCAC)	41.81	41.33
[6]	r(UACAGUCCAUG)<br>d(ATGTCGGGTAC)	45.34	44.07	[6]	r(AACGGUGCGAA)<br>d(TTGCCGCGCTT)	52.98	56.2	[6]	r(CUACGUUGCAA)<br>d(GATGCGACGTT)	39.82	39.81
[6]	r(CGCUUAACUAG)<br>d(GCGAGTTGATC)	33.11	34.11	[6]	r(UAAGAUUGGUA)<br>d(ATTCTAGCCAT)	36.63	32.27	[6]	r(GGAACUUCAUU)<br>d(CCTTGAGGTAA)	37.26	37.18
[6]	r(AGUCUUUCAGG)<br>d(TCAGAGAGTCC)	33.76	37.89	[6]	r(CUAACAUGAGG)<br>d(GATTATACTCC)	32.2	31.21	[6]	r(GUUGAACCUAC)<br>d(CAACTTAGATG)	30.29	28.91
[6]	r(AAUACCCACCG)<br>d(TTATGAGTGGC)	33.09	33.97	[6]	r(AAUGCCGUAUG)<br>d(TTACGACATAC)	31.01	27.77	[6]	r(CAUCGCAGCAA)<br>d(GTAGCATCGTT)	37.4	35.96
[6]	r(AAUGCCGUAUG)<br>d(TTACAGCATAC)	33.04	32.25	[6]	r(AAUAGCUCUGG)<br>d(TTATCAAGACC)	30.96	31.62	[6]	r(UACAGUCCAUG)<br>d(ATGTCAAGTAC)	30.59	30.02
[6]	r(ACGUUCGGAUC)<br>d(TGCAAACCTAG)	37.46	37.72	[6]	r(UGGUCUGUAGA)<br>d(ACCAAACATCT)	31.32	33.27	[6]	r(CUAACAUGAGG)<br>d(GATTTTACTCC)	30.94	31.67
[6]	r(GUUGAACCUAC)<br>d(CAACTTTGATG)	30.33	29.24	[6]	r(AAUACCCACCG)<br>d(TTATGGTTGGC)	32.43	32.75	[6]	r(AAUACCCACCG)<br>d(TTATGTGTGGC)	34.19	36.15
[6]	r(AAUGCCGUAUG)<br>d(TTACGTCATAC)	32.39	28.38	[6]	r(CAUCGCAGCAA)<br>d(GTAGCTTCGTT)	35.97	34.6	[6]	r(AAUGCCGUAUG)<br>d(TTACTGCATAC)	32.86	30.85
[6]	r(AAUAGCUCUGG)<br>d(TTATCTAGACC)	29.56	29.62	[6]	r(UACAGUCCAUG)<br>d(ATGTCATGTAC)	31.07	30.81	[6]	r(ACGUUCGGAUC)<br>d(TGCAATCCTAG)	36.61	36.91
[6]	r(UGGUCUGUAGA)<br>d(ACCATACATCT)	32.39	33.35	[6]	r(CCUAGAGCAUG)<br>d(GGATATCGTAC)	37.74	37.93	[6]	r(CCUAGAGCAUG)<br>d(GGATCTAGTAC)	37.91	37.19
[6]	r(UUCCAGGAAGG)<br>d(AAGGTACTTCC)	43.65	45.23	[6]	r(GUGUAAGUACC)<br>d(CACATTAATGG)	36.32	31.79	[6]	r(CAUCGCAGCAA)<br>d(GTAGAGTCGTT)	38.24	34.19

(continues on next page)

(S1 continued from previous page)

Ref.	Sequence	T_i	T_i'	Ref.	Sequence	T_i	T_i'	Ref.	Sequence	T_i	T_i'
[6]	r(ACGUUCGGAUC)<br>d(TGCAAGACTAG)	35.62	33.32	[6]	r(GUAGGAACAUG)<br>d(CATCATTGTAC)	35.39	32.77	[6]	r(AACGGUGCGAA)<br>d(TTGCAACGCTT)	42.33	44.44
[6]	r(CAGUGACCAAG)<br>d(GTCAATGGTTC)	35.81	33.55	[6]	r(AACGGUGCGAA)<br>d(TTGCCAAGCTT)	40.45	39.67	[6]	r(UGGUCUGUAGA)<br>d(ACCAGAAATCT)	30.57	31.66
[6]	r(CCUAGAGCAUG)<br>d(GGATTTCGTAC)	43.27	43.19	[6]	r(CCUAGAGCAUG)<br>d(GGATCTTGTAC)	44.84	43.88	[6]	r(UUCCAGGAAGG)<br>d(AAGGTTCTTCC)	46.01	49.23
[6]	r(GUGUAAGUACC)<br>d(CACATTTATGG)	38.24	37.17	[6]	r(UCACACGAUAA)<br>d(AGTGTGTTATT)	37.85	36.03	[6]	r(CAUCGCAGCAA)<br>d(GTAGTGTCGTT)	47.98	46.95
[6]	r(ACGUUCGGAUC)<br>d(TGCAAGTCTAG)	44.06	43.71	[6]	r(CUACGUUGCAA)<br>d(GATGTAACGTT)	40.87	38.78	[6]	r(GUAGGAACAUG)<br>d(CATCTTTGTAC)	41.39	42.18
[6]	r(AACGGUGCGAA)<br>d(TTGCTACGCTT)	51.23	52.77	[6]	r(CAGUGACCAAG)<br>d(GTCATTGGTTC)	44.56	44.09	[6]	r(AACGGUGCGAA)<br>d(TTGCCATGCTT)	50.3	50.72
[6]	r(UGGUCUGUAGA)<br>d(ACCAGATATCT)	41.1	41.32								

Table S2. DNA/RNA sequences containing only canonical base pairs used for optimization reference. Shown are the primary structures with the upper strand in $5' \to 3'$ direction, the measured melting temperatures (T_i) from Refs. [4, 5], and their melting temperatures (T_i') predicted with our new parameters.

Id.	Sequence	T_i	T_i'	Id.	Sequence	T_i	T_i'
C1 [4]	r(AAGCGUAG)<br>d(TTCGCATC)	35.71	27.2	C8 [4]	r(UCACCUAG)<br>d(AGTGGATC)	29.09	21.62
C2 [4]	r(UGAGGUAC)<br>d(ACTCCATG)	41.55	30.41	C9 [4]	r(UUGGCACC)<br>d(AACCGTGG)	40.67	34.11
C3 [4]	r(UUAACUGGC)<br>d(AATTGACCG)	41.47	30.36	C10 [5]	r(AGGUAGGU)<br>d(TCCATCCA)	40.48	36.23
C4 [5]	r(GCUCAACCCG)<br>d(CGAGTTGGGC)	52.47	45.45	C11 [5]	r(GGCGAUGAUG)<br>d(CCGCTACTAC)	52.53	47.43
C5 [5]	r(CGAGGAUGGC)<br>d(GCTCCTACCG)	60.54	56.5	C12 [5]	r(GAGACACACC)<br>d(CTCTGTGTGG)	53.71	47.39
C6 [5]	r(GAACUCUGUG)<br>d(CTTGAGACAC)	39.36	34.64	C13 [5]	r(GGACGACG)<br>d(CCTGCTGC)	48.07	42.29
C7 [5]	r(CGCAGUCCAC)<br>d(GCGTCAGGTG)	57.64	46.1				

Table S3. New DNA/RNA sequences containing single mismatches. Shown are the modifications concerning the canonical sequences, the measured melting temperatures (T_i), the difference between the canonical and mismatch sequences measured melting temperatures ($\Delta T_{i,\,\mathrm{can}}$), and the predicted melting temperatures (T_i') in °C.

Ref.	Sequence	Mod.	T_i	$\Delta T_{i,\,\mathrm{can}}$	T_i'	Ref.	Sequence	Mod.	T_i	$\Delta T_{i,\,\mathrm{can}}$	T_i'
N1	r(GAGACACACC)<br>d(CTCTATGTGG)	G → A	31.8	21.91	34.87	N9	r(AAGCGUAG)<br>d(TTCTCATC)	G → T	13.1	22.61	15.97
N2	r(GAGAAACACC)<br>d(CTCTCTGTGG)	C → A<br>G → C	32.3	21.41	35.56	N10	r(GAACUCUGUG)<br>d(CTTGAAACAC)	G → A	12.3	27.06	21.07
N3	r(GAGAAACACC)<br>d(CTCTGTGTGG)	C → A	32.6	21.11	35.5	N11	r(GAACUCUGUG)<br>d(CTTGATACAC)	G → T	14.8	24.56	21.19
N4	r(GAGAUACACC)<br>d(CTCTCTGTGG)	C → U<br>G → C	32.7	21.01	34.98	N12	r(GAACUUUGUG)<br>d(CTTGACACAC)	C → U<br>G → C	14.9	24.46	21.39
N5	r(GAGACACACC)<br>d(CTCTTTGTGG)	G → T	34.3	19.41	35.04	N13	r(GAACUAUGUG)<br>d(CTTGAGACAC)	C → A	15.1	24.26	22.13
N6	r(GAGAGACACC)<br>d(CTCTATGTGG)	C → G<br>G → A	36.6	17.11	40.66	N14	r(GAACUAUGUG)<br>d(CTTGACACAC)	C → A<br>G → C	15.6	23.76	22.1
N7	r(GAGACCCACC)<br>d(CTCTGAGTGG)	A → C<br>T → A	36.7	17.01	38.7	N15	r(GAACUGUGUG)<br>d(CTTGAAACAC)	C → G<br>G → A	16.6	22.76	21.87
N8	r(GAGACCCACC)<br>d(CTCTGTGTGG)	A → C	39.7	14.01	41.39						

Table S4. Seed parameters for Morse potential D in meV. The number of occurrences of each type of mismatched base pair is indicated as n.

Base pair	D	n	Base pair	D	n
dArA	27.8	14	dArC	27.8	15
dArG	27.8	17	dCrA	73.7	20
dCrC	73.7	11	dCrU	73.7	19
dGrA	62.6	20	dGrG	62.6	14
dGrU	62.6	22	dTrC	40.3	16
dTrG	40.3	22	dTrU	40.3	14
dUrG	40.3	4			

Table S5. Seed parameters for harmonic potential k in eV/nm^2. The number of occurrences of each type of nearest-neighbor configuration is indicated as n.

NN	k	n	NN	k	n	NN	k	n	NN	k	n
dArA-dArU	0.9	4	dArA-dCrG	2.8	4	dArA-dGrC	2.8	3	dArA-dTrA	3.1	3
dArC-dArU	0.9	4	dArC-dCrG	2.8	4	dArC-dGrC	2.8	4	dArC-dTrA	3.1	3
dArG-dArU	0.9	4	dArG-dCrG	2.8	4	dArG-dGrC	2.8	4	dArG-dTrA	3.1	5
dArU-dArA	0.9	2	dArU-dArC	0.9	3	dArU-dArG	0.9	4	dArU-dArU	0.9	37
dArU-dCrA	2.8	5	dArU-dCrC	2.8	2	dArU-dCrG	2.8	119	dArU-dCrU	2.8	5
dArU-dGrA	2.8	5	dArU-dGrC	2.8	86	dArU-dGrG	2.8	3	dArU-dGrU	2.8	5
dArU-dTrA	3.1	67	dArU-dTrC	3.1	3	dArU-dTrG	3.1	5	dArU-dTrU	3.1	2
dCrA-dArU	2.6	4	dCrA-dCrG	3.1	6	dCrA-dGrC	1.6	6	dCrA-dTrA	3.1	4
dCrC-dArU	2.6	2	dCrC-dCrG	3.1	4	dCrC-dGrC	1.6	4	dCrC-dTrA	3.1	3
dCrG-dArA	2.6	4	dCrG-dArC	2.6	4	dCrG-dArG	2.6	4	dCrG-dArU	2.6	134
dCrG-dCrA	3.1	5	dCrG-dCrC	3.1	4	dCrG-dCrG	3.1	81	dCrG-dCrU	3.1	6
dCrG-dGrA	1.6	4	dCrG-dGrC	1.6	61	dCrG-dGrG	1.6	4	dCrG-dGrU	1.6	8
dCrG-dTrA	3.1	154	dCrG-dTrC	3.1	4	dCrG-dTrG	3.1	5	dCrG-dTrU	3.1	4
dCrG-dUrG	3.1	2	dCrU-dArU	2.6	3	dCrU-dCrG	3.1	6	dCrU-dGrC	1.6	5
dCrU-dTrA	3.1	5	dGrA-dArU	2.4	4	dGrA-dCrG	2.8	6	dGrA-dGrC	2.6	6
dGrA-dTrA	4.3	4	dGrC-dArA	2.4	4	dGrC-dArC	2.4	5	dGrC-dArG	2.4	5
dGrC-dArU	2.4	74	dGrC-dCrA	2.8	6	dGrC-dCrC	2.8	2	dGrC-dCrG	2.8	74
dGrC-dCrU	2.8	5	dGrC-dGrA	2.6	6	dGrC-dGrC	2.6	83	dGrC-dGrG	2.6	4
dGrC-dGrU	2.6	6	dGrC-dTrA	4.3	182	dGrC-dTrC	4.3	5	dGrC-dTrG	4.3	7
dGrC-dTrU	4.3	4	dGrC-dUrG	4.3	2	dGrG-dArU	2.4	3	dGrG-dCrG	2.8	4
dGrG-dGrC	2.6	4	dGrG-dTrA	4.3	3	dGrU-dArU	2.4	5	dGrU-dCrG	2.8	7
dGrU-dGrC	2.6	5	dGrU-dTrA	4.3	5	dTrA-dArA	0.78	4	dTrA-dArC	0.78	3
dTrA-dArG	0.78	4	dTrA-dArU	0.78	118	dTrA-dCrA	2.5	4	dTrA-dCrC	2.5	3
dTrA-dCrG	2.5	124	dTrA-dCrU	2.5	3	dTrA-dGrA	2.2	5	dTrA-dGrC	2.2	155
dTrA-dGrG	2.2	3	dTrA-dGrU	2.2	3	dTrA-dTrA	2.4	94	dTrA-dTrC	2.4	4
dTrA-dTrG	2.4	5	dTrA-dTrU	2.4	4	dTrC-dArU	0.78	4	dTrC-dCrG	2.5	4
dTrC-dGrC	2.2	5	dTrC-dTrA	2.4	3	dTrG-dArU	0.78	4	dTrG-dCrG	2.5	4
dTrG-dGrC	2.2	8	dTrG-dTrA	2.4	6	dTrU-dArU	0.78	4	dTrU-dCrG	2.5	3
dTrU-dGrC	2.2	4	dTrU-dTrA	2.4	3	dUrG-dCrG	2.5	2	dUrG-dGrC	2.2	2

Table S6. Final optimized average Morse potential depth D and standard deviation $\sigma(D)$ for mismatched DNA/RNA base pairs.

Base pair	D	$\sigma(D)$	Base pair	D	$\sigma(D)$
dArA	1.8	0.9	dArC	3.4	1.3
dArG	4.4	1.0	dCrA	4.0	1.0
dCrC	1.2	0.9	dCrU	2.8	1.2
dGrA	3.4	1.0	dGrG	5.2	0.9
dGrU	6.6	1.1	dTrC	3.8	1.3
dTrG	44.4	1.0	dTrU	3.5	1.4
dUrG	17.0	1.7			

Table S7. Final average harmonic potential coupling constant k and standard deviation $\sigma(k)$ in eV/nm^2 for nearest-neighbor stacking configurations containing a single mismatched base pair.

NN	k	$\sigma(k)$	NN	k	$\sigma(k)$
dArA-dArU	0.0054	0.0031	dArA-dCrG	0.27	0.061
dArA-dGrC	0.016	0.0081	dArA-dTrA	0.63	0.25
dArC-dArU	0.0084	0.0082	dArC-dCrG	5.2	0.47
dArC-dGrC	0.035	0.015	dArC-dTrA	2.0	0.49
dArG-dArU	0.0045	0.0021	dArG-dCrG	9.2	0.61
dArG-dGrC	3.4	0.53	dArG-dTrA	17.0	1.0
dArU-dArA	1.2	0.48	dArU-dArC	0.003	0.0023
dArU-dArG	0.43	0.098	dArU-dCrA	0.84	0.14
dArU-dCrC	0.056	0.07	dArU-dCrU	0.14	0.048
dArU-dGrA	0.85	0.16	dArU-dGrG	0.65	0.23
dArU-dGrU	4.6	1.1	dArU-dTrC	0.004	0.005
dArU-dTrG	7.0	0.52	dArU-dTrU	11.0	1.6
dCrA-dArU	0.0021	0.0024	dCrA-dCrG	3.3	0.33
dCrA-dGrC	0.11	0.037	dCrA-dTrA	2.8	0.51
dCrC-dArU	0.02	0.015	dCrC-dCrG	0.069	0.027
dCrC-dGrC	0.006	0.0031	dCrC-dTrA	0.0049	0.0043
dCrG-dArA	6.3	0.44	dCrG-dArC	5.2	0.49
dCrG-dArG	3.8	0.46	dCrG-dCrA	7.4	0.5
dCrG-dCrC	0.81	0.21	dCrG-dCrU	1.6	0.32
dCrG-dGrA	6.8	0.47	dCrG-dGrG	9.8	0.57
dCrG-dGrU	6.6	0.61	dCrG-dTrC	3.7	0.44
dCrG-dTrG	4.3	0.22	dCrG-dTrU	5.6	0.52
dCrG-dUrG	11.0	0.86	dCrU-dArU	0.0015	0.0012
dCrU-dCrG	3.0	0.39	dCrU-dGrC	1.2	0.26
dCrU-dTrA	0.49	0.33	dGrA-dArU	0.0028	0.0027
dGrA-dCrG	5.1	0.44	dGrA-dGrC	0.57	0.14
dGrA-dTrA	3.6	0.57	dGrC-dArA	0.14	0.053
dGrC-dArC	7.5	0.57	dGrC-dArG	1.2	0.31
dGrC-dCrA	8.8	0.52	dGrC-dCrC	0.76	0.23
dGrC-dCrU	2.3	0.48	dGrC-dGrA	11.0	0.69
dGrC-dGrG	10.0	0.7	dGrC-dGrU	13.0	0.96
dGrC-dTrC	8.5	0.61	dGrC-dTrG	5.6	0.26
dGrC-dTrU	1.3	0.29	dGrC-dUrG	11.0	0.84
dGrG-dArU	0.036	0.018	dGrG-dCrG	7.1	0.61
dGrG-dGrC	1.8	0.33	dGrG-dTrA	1.6	0.49
dGrU-dArU	0.051	0.064	dGrU-dCrG	7.3	0.74
dGrU-dGrC	3.3	0.58	dGrU-dTrA	7.8	1.3
dTrA-dArA	0.0013	0.0011	dTrA-dArC	0.062	0.029
dTrA-dArG	0.095	0.04	dTrA-dCrA	0.092	0.042
dTrA-dCrC	0.001	0.0008	dTrA-dCrU	0.37	0.25
dTrA-dGrA	0.05	0.019	dTrA-dGrG	0.067	0.039
dTrA-dGrU	4.0	0.95	dTrA-dTrC	0.17	0.074
dTrA-dTrG	3.8	0.26	dTrA-dTrU	0.0037	0.0026
dTrC-dArU	0.024	0.018	dTrC-dCrG	2.6	0.3
dTrC-dGrC	0.39	0.13	dTrC-dTrA	1.6	0.49
dTrG-dArU	2.1	0.23	dTrG-dCrG	5.4	0.35
dTrG-dGrC	5.0	0.28	dTrG-dTrA	5.3	0.39
dTrU-dArU	0.016	0.011	dTrU-dCrG	2.1	0.33
dTrU-dGrC	0.013	0.0073	dTrU-dTrA	1.9	0.48
dUrG-dCrG	8.8	0.79	dUrG-dGrC	7.3	0.77

[1] Gary Felsenfeld and Shalom Z. Hirschman. A neighbor-interaction analysis of the hypochromism and spectra of DNA. *J. Mol. Biol.*, 13(2):407–427, September 1965. ISSN 0022-2836. doi:10.1016/s0022-2836(65)80106-1. URL http://dx.doi.org/10.1016/S0022-2836(65)80106-1.

[2] Richard Owczarzy. Melting temperatures of nucleic acids: discrepancies in analysis. *Biophys. Chem.*, 117(3):207–215, 2005.

[3] Carlos A. Plata, Stefano Marni, Amos Maritan, Tommaso Bellini, and Samir Suweis. Statistical physics of DNA hybridization. *Phys. Rev. E*, 103(4), April 2021. ISSN 2470-0053. doi:10.1103/physreve.103.042503. URL http://dx.doi.org/10.1103/PhysRevE.103.042503.

[4] Naoki Sugimoto, Mariko Nakano, and Shu-ichi Nakano. Thermodynamics-structure relationship of single mismatches in RNA/DNA duplexes. *Biochem.*, 39(37):11270–11281, 2000. doi:10.1021/bi000819p.

[5] Norman E. Watkins, William J. Kennelly, Mike J. Tsay, Astrid Tuin, Lara Swenson, Hyung-Ran Lee, Svetlana Morosyuk, Donald A. Hicks, and John SantaLucia. Thermodynamic contributions of single internal rA·dA, rC·dC, rG·dG and rU·dT mismatches in RNA/DNA duplexes. *Nucleic Acids Res.*, 39(5):1894–1902, 2011. doi:10.1093/nar/gkq905. URL https://doi.org/10.1093/nar/gkq905.

[6] Tongjun Xiang, Huibao Feng, Xin hui Xing, and Chong Zhang. Thermodynamic parameters contributions of single internal mismatches in RNA/DNA hybrid duplexes. *bioRxiv*, nov 2022. doi:10.1101/2022.11.25.517909. URL https://doi.org/10.1101/2022.11.25.517909.
